# MicroRNA-152-mediated dysregulation of hepatic transferrin receptor 1 in liver carcinogenesis

**DOI:** 10.18632/oncotarget.6004

**Published:** 2015-10-19

**Authors:** Iryna Kindrat, Volodymyr Tryndyak, Aline de Conti, Svitlana Shpyleva, Thilak K. Mudalige, Tetyana Kobets, Anna M. Erstenyuk, Frederick A. Beland, Igor P. Pogribny

**Affiliations:** ^1^ Division of Biochemical Toxicology, National Center for Toxicological Research, U.S. Food and Drug Administration, Jefferson, AR, USA; ^2^ Office of Regulatory Affairs, Arkansas Regional Laboratory, U.S. Food and Drug Administration, Jefferson, AR, USA; ^3^ Department of Biological and Medical Chemistry, Ivano-Frankivsk National Medical University, Ivano-Frankivsk, Ukraine

**Keywords:** hepatocellular carcinoma, iron metabolism, transferrin receptor 1, microRNA-152, dysregulation

## Abstract

Over-expression of transferrin receptor 1 (TFRC) is observed in hepatocellular carcinoma (HCC); however, there is a lack of conclusive information regarding the mechanisms of this dysregulation. In the present study, we demonstrated a significant increase in the levels of TFRC mRNA and protein in preneoplastic livers from relevant experimental models of human hepatocarcinogenesis and in human HCC cells. Additionally, using the TCGA database, we demonstrated an over-expression of *TFRC* in human HCC tissue samples and a markedly decreased level of microRNA-152 (miR-152) when compared to non-tumor liver tissue. The results indicated that the increase in levels of TFRC in human HCC cells and human HCC tissue samples may be attributed, in part, to a post-transcriptional mechanism mediated by a down-regulation of miR-152. This was evidenced by a strong inverse correlation between the level of TFRC and the expression of miR-152 in human HCC cells (*r* = −0.99, *p* = 4. 7 × 10^−9^), and was confirmed by *in vitro* experiments showing that transfection of human HCC cell lines with miR-152 effectively suppressed *TFRC* expression. This suggests that miR-152-specific targeting of *TFRC* may provide a selective anticancer therapeutic approach for the treatment of HCC.

## INTRODUCTION

Hepatocellular carcinoma (HCC) is the most frequent type of liver cancer and a major contributor to annual cancer mortality rates [1, 2]. While the overall cancer incidence in the United States has steadily declined [3], the incidence of HCC continues to increase [2, 3]. This is evidenced by comprehensive epidemiological data showing that the overall age-adjusted incidence rates of HCC tripled in the United States in the period between 1975 and 2005 [2] and continued to rise in 1990–2009 [3].

HCC arises through a complex multistage process of interconnected events and various molecular, cellular, and metabolic deregulations driven by genetic and epigenetic aberrations. While the main risk factors that account for most HCC cases are well identified (chronic viral hepatitis B and C infection, chemical exposure, alcohol consumption, obesity, and non-alcoholic fatty liver disease), the cellular and molecular processes that mediate HCC development and progression are not fully understood. This signifies a crucial need to understand better the underlying mechanisms of HCC development to prevent the disease and improve its clinical management.

Acquired evidence indicates that cancer [4], including HCC [5, 6], along with various cancer-associated molecular abnormalities, exhibits profound alterations in systemic and intracellular iron homeostasis. Specifically, patients with HCC are characterized by an abnormal iron uptake [7], an excessive accumulation of hepatic iron [5], a prevalence of mutations in the hemochromatosis (*HFE*) gene [8], one of the main mediators of iron metabolism, and a pronounced suppression in the expression of hepcidin (*HAMP*) [9], the central regulator of iron homeostasis in mammals [10]. Importantly, dysregulated iron metabolism has been found not only in both full-fledged rodent and human HCC but also in several chronic liver pathological states associated with the development of HCC, including chronic hepatitis B and C virus infection [11, 12], alcoholic and non-alcoholic fatty liver disease [13–14], and liver fibrosis [15]. While the development of HCC associated with iron overload has been studied extensively, there is a lack of conclusive information to clarify the role of iron metabolism disturbances in liver carcinogenesis that is not caused by iron overload.

On the basis of our previous observations of cancer-related aberrations in intracellular iron homeostasis characterized by an altered expression of the transferrin receptor 1 (*Tfrc*), a main regulator of transferrin-bound iron uptake in mammalian cells during liver carcinogenesis [16], the goal of the present study was to investigate the underlying mechanisms associated with a cancer-related *Tfrc* dysregulation by using *in vitro* and *in vivo* models of liver carcinogenesis. We found substantial up-regulation of TFRC in preneoplastic livers, human liver cancer cell lines, and human HCC tissue samples. Furthermore, we demonstrated that the over-expression of *TFRC* was accompanied by and may be attributed mechanistically to a markedly reduced expression of microRNA-152 (miR-152) in HCC.

## RESULTS

### TFRC and FPN1 proteins and the hepatic iron content in preneoplastic livers

**Figure 1:**
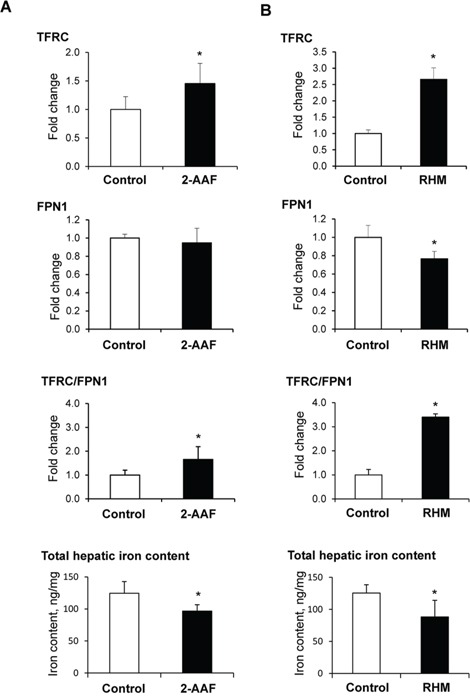
Western blot analysis of TFRC and FPN1 proteins and the hepatic iron content in preneoplastic livers of rats subjected to 2-acetylaminofluorene treatment (A) or a “resistant hepatocyte model” (B) of liver carcinogenesis Data are presented as an average fold change (mean ± S.D., *n* = 5) in the livers of rats undergoing hepatocarcinogenesis relatively to that in control rats, which were assigned *a* value 1. *- Significantly different from the control rats.

Our previous study of 2-acetylaminofluorene (2-AAF)-induced rat hepatocarcinogenesis demonstrated extensive alterations of iron metabolism in preneoplastic livers, characterized by an aberrant expression of genes involved in the maintenance of intracellular iron homeostasis, especially an up-regulation of *Tfrc* and *Fpn1* genes and a down-regulation of *Hamp.* In order to investigate the underlying mechanisms of iron metabolism disturbances during liver carcinogenesis, we first determined the levels of TFRC and FPN1 proteins in the livers of rats undergoing hepatocarcinogenesis. Figure 1 shows that levels of TFRC protein in the preneoplastic livers in rats treated with 2-AAF (Figure 1A) and in rats subjected to a “resistant hepatocyte model” (Figure 1B) were significantly increased, with the magnitude of changes being greater in rats subjected to a more severe “resistant hepatocyte model” of hepatocarcinogenesis. In contrast, levels of FPN1 either did not change (2-AAF model) or decreased (“resistant hepatocyte model”). This resulted in a marked increase of TFRC/FPN1 ratio in preneoplastic livers. However, despite these changes favoring iron uptake, the hepatic iron content in the preneoplastic livers was significantly reduced (Figure 1).

### TFRC expression and the level of intracellular iron in human liver cancer cells

**Figure 2:**
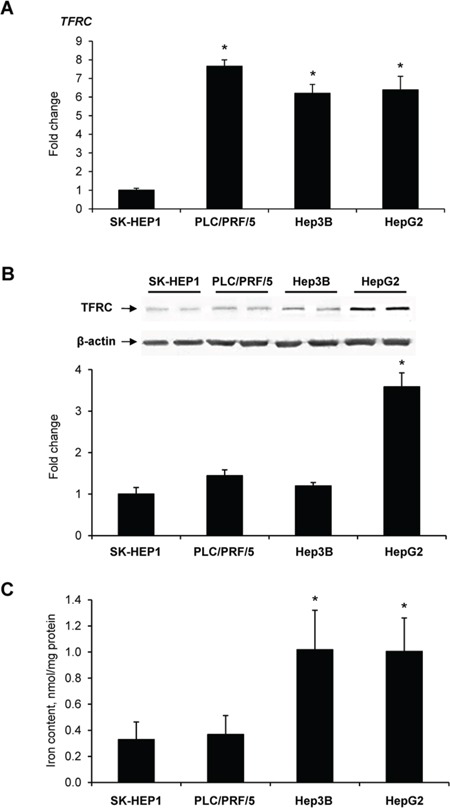
The level of TFRC mRNA (A), TFRC protein (B), and intracellular iron (C) in human liver cancer cells Data are presented as an average fold change (mean ± S.D., *n* = 5) in α-fetoprotein- and EPCAM-positive PLC/PRF/5, Hep3B, and HepG2 cells relatively to that in α-fetoprotein- and EPCAM-negative SK-HEP1 cells. *- Significantly different from SK-HEP1 cells.

To determine further whether or not TFRC alterations found in preneoplastic livers exist also in liver cancer cells, the expression of *TFRC* and level of TFRC protein were investigated in human liver cancer cells *in vitro*. Figure 2 shows (Figure 2A) that human liver cancer cells expressed *TFRC* at a level that varied approximately 6.2- to 7.6-fold, with the lowest expression being found in α-fetoprotein- and EPCAM-negative SK-HEP1 cells as compared to α-fetoprotein- and EPCAM-positive PLC/PRF/5, Hep3B, and HepG2 cells [23, 24]. Since gene expression does not always correlate with the level of a protein encoded by the corresponding gene [25], the level of TFRC was measured in liver cancer cells. The level of TFRC was increased, 3.6-fold, in HepG2 cells only, while not different in SK-HEP1, PLC/PRF/5, or Hep3B cells (Figure 2B). Additionally, HepG2 and Hep3B cells were characterised by 2.9 times greater content of intracellular iron than SK-HEP1 and PLC/PRF/5 cells (Figure 2C).

### Mechanism of TFRC dysregulation in hepatocarcinogenesis

**Figure 3:**
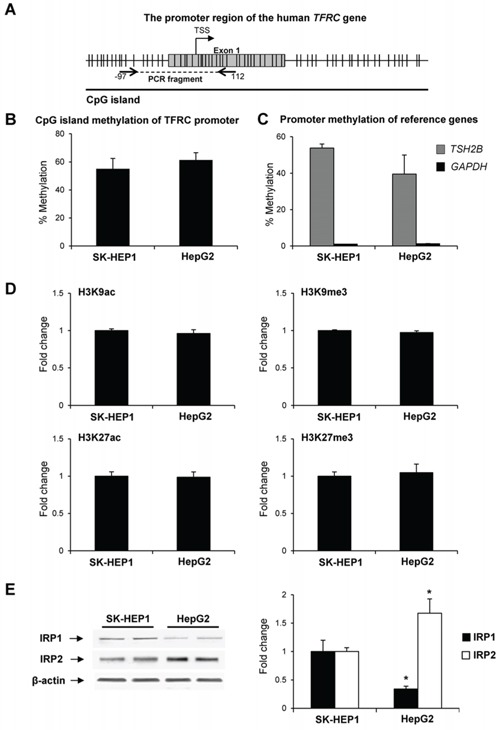
The status of cytosine DNA methylation and histone modifications of the TFRC gene in human liver cancer cells **A.** Diagram of the 5′-region of the *TFRC* gene. **B.** The extent of CpG island methylation in the *TFRC* promoter in SK-HEP1 and HepG2 cells. **C.** The extent of methylation of the *TSH2B* and *GAPDH* genes in SK-HEP1 and HepG2 cells. The *TSH2B* and *GAPDH* genes were used as methylated and un-methylated reference control genes and showed a marked difference in the extent of the methylation between SK-HEP1 and HepG2 cells, whereas no differences in the extent of *TFRC* methylation were found in these cell lines. **D.** Chromatin immunoprecipitation assay of histone H3K9ac, H3K9me3, H3K27ac, and H3K27me3 in the *TFRC* promoter. **E.** Western blot analysis of IRP1 and IRP2 in SK-HEP1 and Hep2G cells. Data are presented as an average fold change in HepG2 cells relatively to that in SK-HEP1 cells.

It is well-established that the expression of the *TFRC* gene is regulated at transcriptional and post-transcriptional levels [26, 27]. In light of this, the role of epigenetic mechanisms in *TFRC* dysregulation at the transcriptional level in human liver cancer cells was investigated. Figure 3 shows that there were no differences in the level of *TFRC* CpG island methylation (Figure 3B) or in the promoter enrichment by histone H3K9ac, H3K9me3, H3K27ac, and H3K27me3 (Figure 3D) between SK-HEP1 and HepG2 cells, two cell lines characterized by vast differences in *TFRC* expression.

At the post-transcriptional level, the regulation of *TFRC* occurs through an interaction of iron-regulatory proteins 1 and 2 (IRP1 and IRP2), with the iron-regulatory elements located in the 3′-utranslated region (3′-UTR) of the *TFRC* mRNA [28]. Figure 3E shows that the level of IRP1 protein in HepG2 cells was markedly lower, while the level of IRP2 protein was substantially greater as compared to SK-HEP1 cells. Similarly, an increased level of IRP2 was found in the preneoplastic livers in rats treated with 2-AAF or subjected to a “resistant hepatocyte model” of hepatocarcinogenesis (data not shown).

The presence of *TFRC*-expression controlling elements in the 3′-UTR suggests that the expression of *TFRC* may be mediated by microRNAs (miRNAs). In order to investigate this mechanism of *TFRC* dysregulation at the post-transcriptional level, three public databases for target gene prediction, PicTar (http://pictar.mdc-berlin.de), DIANA-TarBase, version 7 (http://www.microrna.gr/tarbase), and TargetScan, version 6.2 (http://www.targetscan.org), were used to screen for miRNAs with the potential to bind to the 3′-UTR of *TFRC*. The results of *in silico* screening analysis demonstrated that several miRNAs, including miR-152, miR-194, and miR-320, could target the 3′-UTR of *TFRC* mRNA. Hence, the expression of these miRNAs was investigated in human liver cancer cells.

**Figure 4:**
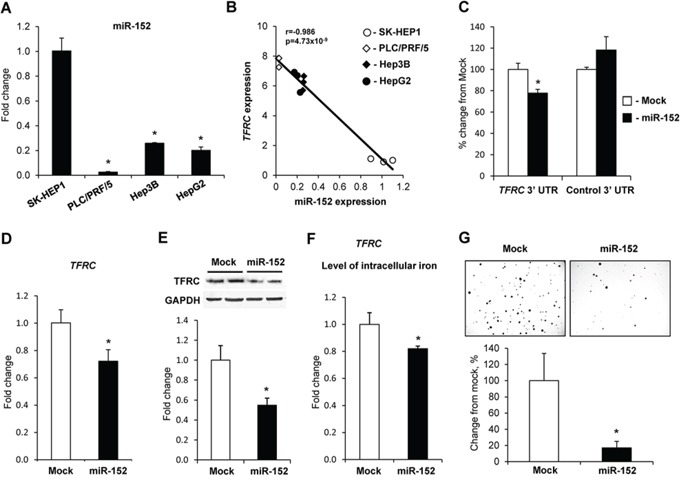
Regulation of *TFRC* expression by miR-152 **A.** The expression of miR-152 in human liver cancer cells. Data are presented as an average fold change (mean ± S.D., *n* = 5) in α-fetoprotein- and EPCAM-positive PLC/PRF/5, Hep3B, and HepG2 cells relatively to that in α-fetoprotein- and EPCAM-negative SK-HEP1 cells. *- Significantly different from SK-HEP1 cells. **B.** Correlation plot of miR-152 and TFRC expression in human liver cancer cells. **C.** Inhibition of *TFRC* expression in the 3′-UTR luciferase reporter assay after transfection of the SK-HEP1 cells with miR-152 or a negative control. **D.** Ectopic up-regulation of miR-152 inhibited *TFRC* expression and reduced level of TFRC protein **E.** in HepG2 cells. *- Significantly different from HepG2 cells transfected with scrambled RNA oligonucleotide. **F.** Effect of ectopic up-regulation of miR-152 in HepG2 on the level of intracellular iron and **G.** soft agar colony formation. *- Significantly different from HepG2 cells transfected with scrambled RNA oligonucleotide.

The expression of miR-152 in α-fetoprotein-positive PLC/PRF/5, Hep3B, and HepG2 cells was considerably lower than in α-fetoprotein-negative SK-HEP1 cells (Figure 4A) and inversely correlated with the expression of *TFRC* (Figure 4B). In contrast, the expression of miR-194 in PLC/PRF/5, Hep3B, and HepG2 cells was substantially greater than in SK-HEP1 cells and there was no major difference in the level of miR-320 among cell lines (Supplementary Figure 1). To test whether or not miR-152 directly targets *TFRC*, constructs retaining the core region of the *TFRC* 3′-UTR that harbors the putative binding site for miR-152 or mutated sequence in the miR-152 binding site were constructed and then co-transfected together with miR-152 into SK-HEP1 cells. Figure 4C shows that miR-152 efficiently suppressed luciferase activity by 22.3%. In contrast, miR-152 did not affect luciferase activity in cells transfected with the *TFRC* 3′-UTR that harbored the mutated miR-152 binding site (data not shown). To confirm further the involvement of miR-152 in the regulation of *TFRC* at the post-transcriptional level, HepG2 cells were transfected with miR-152, miR-194, miR-320 microRNA mimics, or scrambled RNA oligonucleotide. Transfection with miR-152 efficiently down-regulated TFRC at both mRNA and protein levels (Figure 4D and 4E), while transfection with either miR-194 or miR-320 did not (data not shown). Additionally, transfection of HepG2 cells with miR-152 significantly reduced the level of intracellular iron (Figure 4F) and colony formation (Figure 4G).

### Expression of TFRC and miR-152 in human HCC

**Figure 5:**
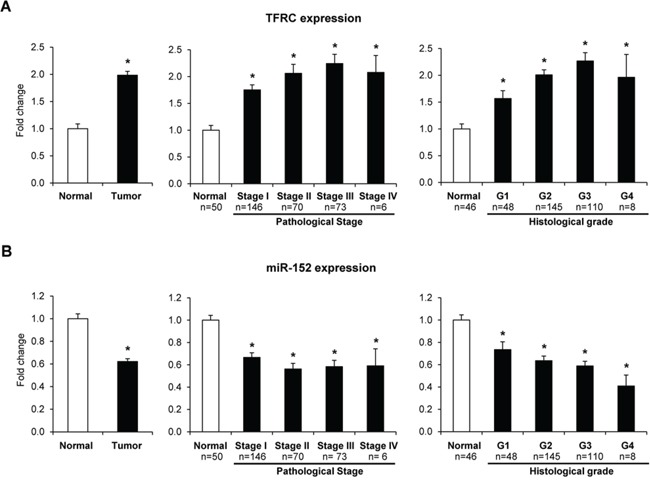
Expression of *TFRC* and miR-152 in human HCC samples Gene expression *TFRC* and miR-152, and clinical and tumor pathological data were extracted from TCGA. * -Significantly different from normal liver samples.

The expression levels of *TFRC*, miR-152, and miR-194 in human HCC tissue samples and normal liver samples were extracted from the TCGA database. Figure 5 shows that the expression of *TFRC* in HCC tissue samples (*n* = 345) was significantly greater as compared to normal livers and increased with the progression of HCC. In contrast, the level of miR-152 was markedly reduced in HCC tissue samples, with the values being significantly lower at each tumor stage of HCC progression, and inversely correlated with the *TFRC* expression (*p* = 0.0078). There were no differences in the expression of miR-194 between normal and HCC samples (Supplementary Figure 2).

## DISCUSSION

HCC is a complex and highly prevalent lethal cancer worldwide. Rapid progression, insufficient knowledge of cellular and molecular processes associated with HCC development, and the asymptomatic nature of early disease are major challenges for the detection and clinical management of HCC. It is commonly accepted that a better understanding of pathways and processes involved in the pathogenesis of HCC may lead to a better prediction of disease, the identification of novel molecular targets, and provide the opportunity for the development of more appropriate and curative treatment approaches.

In the present study, we investigated the role and mechanisms of TFRC dysregulation in liver carcinogenesis by using two rat models of hepatocarcinogenesis, human liver cancer cell lines, and human HCC gene expression data. The results of this study demonstrate that the level of TFRC was substantially greater in the preneoplastic livers of rats undergoing carcinogenesis, in α-fetoprotein- and EPCAM-positive PLC/PRF/5, Hep3B, and HepG2 human liver cells, and in human HCC tissue samples. Previously, up-regulation of TFRC has been observed in both rodent and human liver carcinogenesis. Specifically, Pascale *et al*. [30] and Takahashi *et al*. [31], using two different models of rat hepatocarcinogenesis, demonstrated an over-expression of TFRC in preneoplastic glutathione-*S*-transferase placental form (GSTP)-positive foci. More importantly, the expression of TFRC was increased in parallel with the stage of carcinogenesis. A separate, but similar, study by Mizukami *et al*. [32] confirmed this finding and suggested that up-regulation of TFRC may be involved in the formation and progression of GSTP-positive preneoplastic lesions. This suggestion is supported by the findings of the present study and by a recent report demonstrating an up-regulation of *TFRC* in human HCC samples as compared to adjacent not-tumor tissue and that TFRC over-expression was significantly associated with serum levels of α-fetoprotein [33].

Several mechanisms may contribute to the dysregulation of TFRC. First, it has been suggested that over-expression of TFRC may be a response to intracellular iron deficiency during liver carcinogenesis [33]. The results of the present study showing up-regulation of TFRC in preneoplastic liver with decreased hepatic iron content support this suggestion. Second, it has been shown that under the iron-limited conditions, IRP1 and, especially, IRP2 increase the expression of TFRC; however, IRP2 has been shown to be the chief physiologic regulator of iron metabolism [28, 34]. This was evidenced by the fact that the over-expression of IRP2 is associated with up-regulation of TFRC [35]. Additionally, it has been demonstrated that the IRP2 binding activity is selectively activated by low iron, positively correlated with a TFRC expression [36], and the induction of IRP2 promoted tumor growth [37], while up-regulation of IRP1 exhibited a tumor suppressive effect [38]. These findings are in good agreement with our observations of up-regulation of TFRC and IRP2 and down-regulation of IRP1 in α-fetoprotein- and EPCAM-positive HepG2 cells as compared α-fetoprotein-negative SK-HEP1 cells. Third, Du *et al*. [39] have demonstrated a direct role for the iron regulatory hepatic hormone hepcidin in the inhibition of *TFRC* expression. Importantly, a reduction of hepcidin expression and an over-expression of *TFRC* during experimental liver carcinogenesis and in human HCC has been reported in several studies [9, 16, 40]. Finally, several transcription factors, including MYC and STAT5, are involved in regulation of TFRC [25, 41, 42]. Specifically, O'Donnell *et al*. [42] demonstrated that MYC directly binds to the conserved canonical E-box sequence (CACGTG) in intron1 of the *TFRC* gene and activates its expression. Furthermore, the latter study demonstrated that enforced up-regulation of TFRC facilitates cell proliferation, promotes tumorigenesis of Rat1a-myc cells, and increases the rate of tumor formation *in vivo*.

The results of the present study showed an additional mechanism of the regulation of *TFRC* expression at post-transcriptional level mediated by miR-152, especially in full-fledged human HCC cells. It has been reported previously that three miRNAs, miR-31, miR-210, and miR-320, in addition to miR-152, may target *TFRC* [43–45]; however, considering a high degree of miRNA tissue specificity, the biological significance of any given miRNA-mRNA interaction should be evaluated in a specific target tissue context. The results of the present study demonstrate that miR-152, in addition to miR-31, miR-210, and miR-320 miRNAs, targets *TFRC* directly, evidenced by functional miR-152-TFRC analyses and an inverse correlation between markedly decreased miR-152 level and *TFRC* up-regulation in human HCC cells and HCC tissue samples. This finding is in a good agreement with a previous report that showed down-regulation of miR-152 in human HBV-related HCC [46]. Likewise, Chen *et al*. [47] have reported a significant down-regulation of miR-320 in liver tumors.

Another important finding of this study is that an inhibition of *TFRC* expression by enforced up-regulation of miR-152 in human HepG2 cells was associated with a reduction of the level of intracellular iron and tumorigenic potential of the miR-152-transfected HepG2 cells. It has been reported previously that modulation of iron availability in cancer cells by the use of iron chelating agents or through transferrin receptor-targeted treatments exhibits potent anti-tumor activity, including an anti-HCC effect. Specifically, treatment of liver cancer cells with iron chelators significantly decreased the cellular iron content and exhibited strong anti-proliferative and tumor-growth suppressing effects [48]. Similarly, inhibition of TFRC, either via knocking down of *TFRC* or by anti-TFRC antibodies, induced iron depletion and cell death [29, 49]. In light of this, the results of the present study suggest that targeting of *TFRC* by miR-152 in HCC may be an attractive therapeutic approach for the treatment of HCC.

In summary, the data presented herein indicate that over-expression of *TFRC* may be responsible for profound cancer-associated abnormalities in cellular iron metabolism during liver carcinogenesis. Mechanistically, the up-regulation of *TFRC* may be attributed to a markedly reduced expression of miR-152. Future studies are needed to determine the safety and therapeutic efficiency of correcting cancer-associated aberrations in iron homeostasis by modulating iron availability through the use of miRNA-based transferrin receptor targeting. However, the results of this study provide support for developing of treatment strategies for HCC through corrections of iron metabolism disturbances.

## MATERIALS AND METHODS

### *In vivo* rat models of rat hepatocarcinogenesis

Two well-established models of rat hepatocarcinogenesis, a 2-acetylaminofluorene (2-AAF)-induced model [17] and a “resistant hepatocyte model” [18], which recapitulate the development of human HCC [19], were used in this study. The in-life portion of this study, tissue collection protocols, and results of histopathological analyses, are detailed in Bagnyukova *et al*. [20] and Kuroiwa-Trzmielina *et al*. [21]. Briefly, in 2-AAF model of liver carcinogenesis male Sprague-Dawley rats were fed diet containing 0.02% of 2-AAF for 24 weeks. In the “resistant hepatocyte model”, male Wistar rats received a single intraperitoneal injection of *N*-nitrosodiethylamine (DEN; 200 mg/kg body weight), following by a treatment with 2-AAF (20 mg/kg body weight) for four consecutive days, then were subjected to a 2/3 partial hepatectomy, and two additional 2-AAF treatments. Rats were sacrificed at 6 weeks after DEN initiation.

### Cell lines and cell culture

SK-HEP1, PLC/PRF/5, Hep3B, and HepG2 human liver cancer cell lines were obtained from the American Type Culture Collection (ATCC, Manassas, VA) and maintained according to ATCC's recommendations. Cells were seeded at density 0.5 × 10^6^ viable cells per 100 mm plate, and the media was changed every other day for 4 days. The cells were scrapped onto ice, washed in phosphate-buffered saline (PBS), and immediately frozen at −80°C for subsequent analyses.

### RNA extraction and quantitative reverse transcription-PCR

Total RNA was extracted from liver tissues and cells using miRNeasy Mini kits (Qiagen, Valencia, CA) according to the manufacturer's instructions. Total RNA (2 μg) was reverse transcribed using random primers and High Capacity cDNA Reverse Transcription kits (Life Technologies, Carlsbad, CA) according to the manufacturer's protocol and gene expression was determined by quantitative reverse transcription PCR (qRT-PCR) using TaqMan gene expression assays (Life Technologies). Each sample was analyzed in triplicate. The relative amount of each mRNA transcript was determined using the 2^−ΔΔCt^ method.

### qRT-PCR analysis of microRNA expression

The level of miR-152, miR-194, and miR-320 in human liver cancer cells was determined by qRT-PCR using TaqMan miRNA assays (Life Technologies).

### Western blot analysis of protein levels

Liver tissue and cell lysates were prepared as described previously [16]. Extracts containing equal quantities of proteins were separated by SDS-PAGE on 8–15% polyacrylamide gels and transferred to PVDF membranes. Membranes were probed with primary antibodies against transferrin receptor 1 (TFRC, TFR1, CD71; 1:500; Santa Cruz Biotechnology, Santa Cruz, CA), ferroportin (FPN1, MTP1; 1:1000; Alpha Diagnostic International Inc., San Antonio, TX), iron-regulatory proteins 1 (IRP1; 1:500; Santa Cruz Biotechnology), and iron-regulatory proteins 2 (IRP2; 1:500; Santa Cruz Biotechnology). IRDye^®^ 800 conjugated secondary anti-rabbit and anti-goat antibodies (1:15000; LI-COR Biosciences, Lincoln, NE) were used for visualization. Blots were scanned and analyzed with an Odyssey CLx Infrared Imaging System (LI-COR Biosciences). Equal protein loading was confirmed by immunostaining against GAPDH (1:5000; Sigma-Aldrich, St. Louis, MO).

### Measurement of hepatic iron content by inductively coupled plasma mass spectrometry

Inductively coupled plasma mass spectrometry (ICP-MS) was used to measure the total hepatic iron content. Briefly, microwave dissolution of the liver samples was performed with 4.0 mL of concentrated HNO_3_ using 100 mg of tissue, followed by quantitative transfer and dilution using 2% HNO_3_. Tissue microwave dissolution was accomplished by application of up to 1600 W power, 200°C for 35 min utilizing a Microwave-Accelerated Reaction System Model MARS-X (CEM Corporation, Matthews, NC). The iron content was determined with an Agilent 8800 Inductively Coupled Plasma mass spectrometer (Santa Clara, CA), utilizing the ^56^Fe isotope. ^45^Sc, at 100 ng/mL, was used as an internal standard. Helium collision cell gas was used for the elimination of argon oxide (^40^Ar^16^O^+^) poly atomic interferences.

### Quantitation of intracellular iron by the colorimetric ferrozine assay

The intracellular iron content of the human liver cancer cells was determined by colorimetric ferrozine-based assay as detailed in Riemer *et al*. [22].

### Methylated DNA immunoprecipitation analysis

Methylated DNA immunoprecipitation (MeDIP) was performed with MethylMiner Methylated DNA Enrichment kits (Invitrogen, Carlsbad, CA) according to the manufacturer's instructions. The methylation status of the CpG island located within the promoter/first exon region of the *TFRC* gene was determined by quantitative PCR (qPCR) of DNA from immunoprecipitates and unbound DNA using forward 5′-GCAGGATGAAGGGAGGACAC-3′ and reverse 5′-GCGATCTGTCAGAGCACCTC-3′ primers.

### Chromatin immunoprecipitation assay

Formaldehyde cross-linking and ChIP assays with primary antibodies against histone H3K9ac, H3K9me3, H3K27ac, and H3K27me3 (Abcam, Cambridge, MA) were performed by using a Chromatin Immunoprecipitation Assay kit (Millipore Corporation, Billerica, MA). Purified DNA from immunoprecipitates and input DNA were analyzed by qPCR with primers for the promoter/first exon region of the *TFRC* gene. The results were normalized to the amount of input DNA and presented as fold change for each DNA in HepG2 cells relative to those in SK-HEP1 cells.

### TFRC 3′-UTR luciferase reporter assay

A luciferase miRNA 3′-UTR target vector carrying the predicted miR-152 binding site in the *TFRC* 3′-UTR, miRNA 3′-UTR target vector harboring two base mutations in the predicted miR-152 binding site in the *TFRC* 3′-UTR, and miRNA 3′-UTR target control vector were constructed and purchased from GeneCopoeia Inc. (Rockville, MD). To assess the direct interaction between miR-152 and the *TFRC* 3′-UTR, the luciferase constructs (1 μg) and the 80 nM of miR-152 mimic (Life Technologies) were co-transfected into SK-HEP1 cells, which were seeded in 6-well plates (1 × 10^6^ cells/transfection) using Lipofectamin™ 3000 transfection reagent (Life Technologies) following the manufacturer's recommendations. SK-HEP1 cells transfected with miR™ Negative Control #1 (Life Technologies) served as the control. Forty-eight hours after the transfection, cells were harvested by mild trypsinization, washed in PBS and the luciferase activity was measured using a Luc-Pair Duo-Luciferase Assay Kit 2.0 (GeneCopoeia Inc.) according to the manufacturer's instructions.

### Transfection of HepG2 cells with microRNA mimics

HepG2 cells were seeded in 100 mm dishes at a density of 1 × 10^6^ cells/dish, and transfected with 20 nM of either miR-152, miR-194, or miR-320 microRNA mimics (Life Technologies), in three independent replicates, using Lipofectamin™ 2000 transfection reagent (Life Technologies) according to the manufacturer's instructions. HepG2 cells transfected with scrambled RNA oligonucleotide served as the control. Seventy-two hours post-transfection, adherent cells were harvested by mild trypsinization and the viability of cells was monitored with an MTT test. The cells were then re-seeded and the transfection was repeated. Seventy-two hours after the second transfection, adherent cells were harvested by mild trypsinization, washed in PBS, and the viability of cells was again determined. The cells were then immediatelly frozen at −80°C for subsequent analyses. The experiments were repeated twice, and each cell line tested in triplicate.

### Soft agar colony formation assay

HepG2 cells (10 × 10^3^ cells), transfected with 20 nM of either miR-152 mimic or scrambled RNA oligonucleotide, were seeded onto 0.7% noble agar in growth media. After 20 days of growth, colonies were stained with 0.005% crystal violet, examined by light microscopy using an Olympus CK2 phase contrast inverted microscope (Olympus America Inc., Center Valley, PA), and digitally photographed at identical exposure settings.

### Retrieval of data from online database

Gene expression data for *TFRC*, miR-152, and miR-194, and clinical and tumor pathological data were extracted as .txt files from The Cancer Genome Atlas database (TCGA; http://cancergenome.nih.gov).

### Statistical analyses

Results are presented as mean ± S.D. Data were analyzed by one-way analysis of variance (ANOVA), with pair-wise comparisons being made by the Student-Newman-Keuls method. When necessary, the data were natural log transformed before conducting the analyses to maintain a more equal variance or normal data distribution. Pearson product-moment correlation coefficients were used to determine the strength of association between levels of *TFRC* mRNA and the expression of miR-152. *P*-values <0.05 were considered significant.

## SUPPLEMENTARY FIGURES


