# Supplementary material for: MicroRNA-152-mediated dysregulation of hepatic transferrin receptor 1 in liver carcinogenesis
Source: Oncotarget. 2015 Oct 19;7(2):1276–87. doi: 10.18632/oncotarget.6004 (PMC4811459; doi:10.18632/oncotarget.6004)
Supplement: Supplementary file 1 [file oncotarget-07-1276-s001.pdf]

SUPPLEMENTARY FIGURES

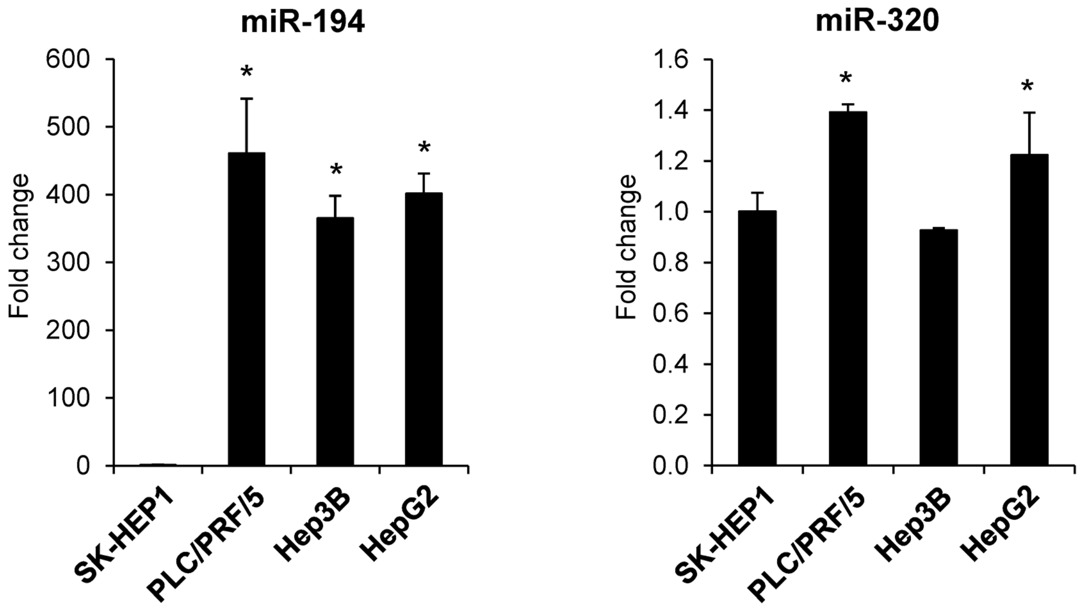

Supplementary Figure S1: The expression of miR-152 in human liver cancer cells. Data are presented as an average fold change (mean ± S.D., $n = 5$) in α-fetoprotein- and EPCAM-positive PLC/PRF/5, Hep3B, and HepG2 cells relatively to that in a–fetoprotein- and EPCAM-negative SK-HEP1 cells. *- Significantly different from SK-HEP1 cells.

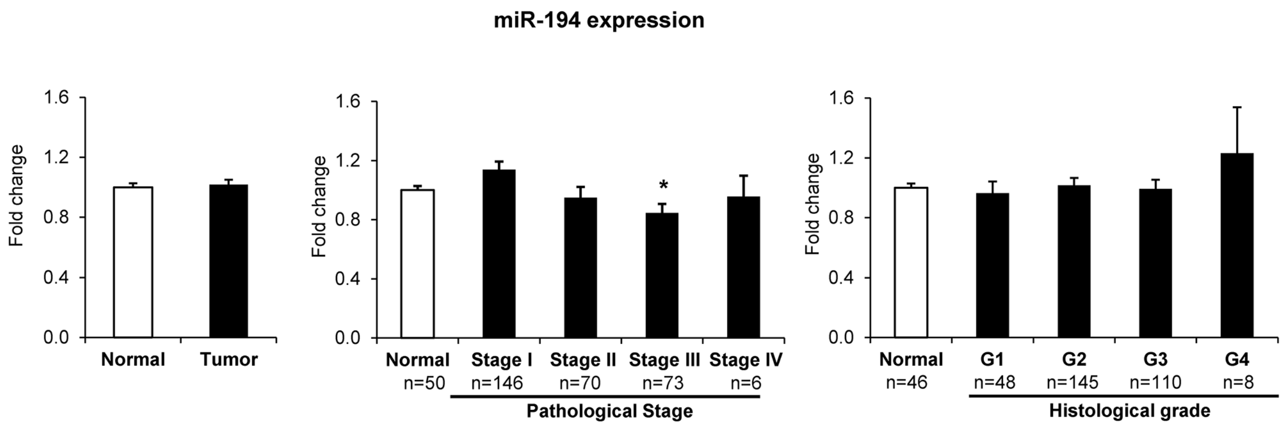

Supplementary Figure S1: Expression of miR-194 in human HCC samples. miR-194 expression and clinical and tumor pathological data were extracted from The Cancer Genome Atlas database (TCGA; http://cancergenome.nih.gov).
